# An Adaptive Time-Varying Impedance Controller for Manipulators

**DOI:** 10.3389/fnbot.2022.789842

**Published:** 2022-03-18

**Authors:** Xu Liang, Tingting Su, Zhonghai Zhang, Jie Zhang, Shengda Liu, Quanliang Zhao, Junjie Yuan, Can Huang, Lei Zhao, Guangping He

**Affiliations:** ^1^Department of Mechanical and Electrical Engineering, North China University of Technology, Beijng, China; ^2^State Key Laboratory of Management and Control for Complex Systems, Institute of Automation, Chinese Academy of Sciences, Beijing, China; ^3^Beijing Aerospace Measurement & Control Technology Co., Ltd, Beijing, China

**Keywords:** adaptive, intelligent control, time-varying, human–robot interaction, MRAC

## Abstract

Aiming at the situation that the structural parameters of the general manipulators are uncertain, a time-varying impedance controller based on model reference adaptive control (MRAC) is proposed in this article. The proposed controller does not need to use acceleration-based feedback or to measure external loads and can tolerate considerable structure parameter errors. The global uniform asymptotic stability of the time-varying closed-loop system is analyzed, and a selection approach for control parameters is presented. It is demonstrated that, by using the proposed control parameter selection approach, the closed-loop system under the adaptive controller is equivalent to an existing result. The feasibility of the presented controller for the general manipulators is demonstrated by some numerical simulations.

## 1. Introduction

The control issues of a multi-degree-of-freedom (multi-DOF) mechanical system with force and motion task constraints are significant for many advanced practical applications, such as minimally invasive surgeries (Burgner-Kahrs et al., 2015), rehabilitation nursing (Jutinico et al., 2017; Ansari et al., 2018), *in-situ* inspection, and machining for the repair of aeroengine parts (Dong et al., 2017; Su et al., 2020), life rescues (McMahan et al., 2006), teleoperation based on haptic interfaces (Sharifi et al., 2016), etc. The operation tasks with force and motion constraint include force-position approximately decoupled operation tasks and more general force-position coupled operation tasks. With regard to the operation task with decoupling force and motion constraint, the closed-loop control system can be stabilized through hybrid force/motion control strategies (Yip and Camarillo, 2016). As to the task with coupling force and motion constraints, in general, an impedance controller has to be utilized to track the time-varying trajectories of the constraint task (Kronander and Billard, 2016). At present, most researches focus on the invariant impedance control of the robot system. The adjustment range of the manipulator's dynamic characteristics under the invariant impedance controller is limited, and it can only complete some rough human-machine cooperation/coordination tasks. For mechanical assembly tasks, especially for those relatively precise assembly operations, such as bearing press-mounting and quantitative fastening of screws/nuts, it is necessary to accurately control the position and pose of the end-effector in the direction of force, as well as the force/torque during the operations. Therefore, the application of invariant impedance control is relatively limited, and the time-varying impedance control has important practical application requirements in engineering tasks. Since the time-varying impedance control has extensive and important application requirements in complex systems or high-level applications such as the universal operation of industrial robots, the interactive motion of rehabilitation robots, human–machine fusion control of exoskeleton robots, telepresence teleoperation robots, etc., in recent years, scholars have conducted related research on time-varying impedance control. The time-varying impedance closed-loop system is a kind of time-varying dynamic system, and it is difficult to analyze its global or large range asymptotic stability. The integration of robotics and artificial intelligence promotes the development of controllers under time-varying operation tasks (Su et al., 2018; Wu et al., 2021a,b). The theoretical and practical research of artificial intelligence control methods based on fuzzy control, neural network and other theories have been carried out internationally for more than 30 years (Deng et al., 2021a,b; Wu et al., 2021c,e). Artificial intelligence control methods often use large-scale inference rule bases or network structures with a large number of nodes and layers in order to ensure their large-scale effectiveness. Due to their good learning ability, artificial intelligence methods are often used in the cognitive science of human–machine interaction systems (Wu et al., 2020b, 2021d).

In practical engineering applications, the control systems often encounter comprehensive characteristics such as strong non-linearity, uncertainty, and time-varying parameters (Liu et al., 2020; Liang et al., 2021b), which will affect the stability of the system. Because the accurate dynamic modeling of the robot system is rather hard, which brings difficulties to the control law design of the system and reduces the dynamic characteristics of the closed-loop system, it is difficult for the robot to achieve high-quality practical applications. Adaptive control and its improvement (Tong et al., 2020; Liu et al., 2021b), sliding mode control and its improvement (Zhai and Xu, 2021), non-linear feedback control and its improvement, observers and its improvement (Liang et al., 2021a; Li et al., 2021; Liu et al., 2021a), and other methods (Yang et al., 2021) can be used to solve this problem. In literature (Li, 2021), a novel command filter adaptive tracking controller is designed to achieve asymptotic tracking for a class of uncertain non-linear systems with time-varying parameters and uncertain disturbances by introducing a smooth function with positive integrable time-varying function to compensate the unknown time-varying parameters and uncertain disturbances. In this article, we study adaptive time-varying impedance controllers. In recent years, adaptive impedance control problems have attracted the attention of many scholars due to the wide and different application requirements (Xu et al., 2011; Jamwal et al., 2017), such as the relevant developments about haptic interfaces (Sharifi et al., 2016), upper/lower limb rehabilitation robots (Li et al., 2017; Liu et al., 2017), robotic exoskeleton systems (Hussain et al., 2013), and so on (Wu et al., 2020a; Deng et al., 2021b). At present, most research studies on adaptive impedance control are actually focused on online “impedance planning,” which means online searching for a target impedance profile for the purpose of improving the application effects of robots. The stability issues of the time-varying closed-loop systems with regard to the target impedance profile are not analyzed except few works (Ferraguti et al., 2013; Kronander and Billard, 2016). In some application-oriented research studies, experiments are always used to demonstrate the stability of the controlled plants (Hamedani et al., 2019; Pena et al., 2019; Perez-Ibarra et al., 2019). However, demonstrating the stability of an adaptive impedance control system by experiments is commonly task-depended, and different operating tasks require different experiments to verify the stability of the system. Therefore, an analysis or control method that can ensure the stability of the time-varying impedance control system is required. To this end, the literature (Kronander and Billard, 2016) and (Ferraguti et al., 2013) addressed this issue. Through an in-depth analysis of the method presented in Ferraguti et al. (2013), the literature (Kronander and Billard, 2016) presented the stability conditions for the variable damping and stiffness system, and the proposed stability conditions do not rely on the controlled plant's states. The benefit of the stability conditions is that they can be verified offline before performing a task. However, this approach has two main shortcomings: (1) accurate dynamics model of the controlled plant is needed in the controller; and (2) measurement of external loads or joint accelerations is required in the controller.

In this article, aiming at the above two problems, a globally uniform stability condition is proposed in which the variable damping and stiffness are independent of the state of the robot. As we all know, the closed-loop system under an adaptive impedance controller is actually a time-varying dynamic system, and it is also a complex non-linear system, which makes it difficult to design the controller. To be specific, the main contribution of this article is summarized as follows:

1. In this article, we use variable damping and stiffness control to adjust damping and stiffness parameters to improve compliant operation performance and use adaptive control to adjust the parameters to achieve the stability of the system when the system parameters are disturbed.
2. Under the frame of model reference adaptive control (MRAC), the rigorous canonical reduction form of the dynamics of the general robot system can be transformed into linear or special non-linear “recursive canonical form.” By using the recursive canonical expression and the design method of the time-varying impedance controller of the linear system, the analytical expression of the parameter adaptive regulation law can be obtained, and the time-varying impedance controller with parameter adaptive characteristics can be designed.
3. The time-varying impedance controller is reconstructed under the frame of MRAC, and the stability condition given in Kronander and Billard (2016) remains unchanged. Therefore, the stability condition under the adaptive control frame is still state independent, while the above two shortcomings are eliminated.

The remainder of this article is organized as follows. Section 2 presents the stability condition provided by Kronander and Billard (2016), since it does not depend on any controllers. Section 3 presents our main contributions, the time-varying impedance controller based on MRAC and a control parameter selection approach. As an example, the method is tested through an uncertain planar 2R manipulator in section 4. Section 5 gives the conclusions.

## 2. Existing Time-varying Impedance Controller for Manipulators

In general, the dynamic equation of the manipulators has the following form


(1) $$\begin{array}{l} M\left(\Theta \right)\ddot{\Theta }+C\left(\Theta ,\dot{\Theta }\right)\dot{\Theta }+N\left(\Theta \right)-\tau_{e}=\tau_{a} \end{array}$$

where Θ ∈ ℜ^*n*^ represents the generalized coordinates of the manipulator in configuration space, *M*(Θ) ∈ ℜ^*n*×*n*^ represents the inertial matrix of the system, $C\left(\Theta ,\dot{\Theta }\right)\dot{\Theta }\in {ℜ}^{n}$ represents centrifugal and Coriolis torque vector, *N*(Θ) ∈ ℜ^*n*^ represents the gravity and elastic force vector, $\tau_{e}\in {ℜ}^{n}$ represents an equivalent torque caused by the external forces, while $\tau_{a}\in {ℜ}^{n}$ represents the actuation torque.

For time-varying impedance control issues, the closed-loop target dynamic equation of a manipulator can be given as follows


(2) $$\begin{array}{l} H\ddot{\overset{-}{\Theta }}+D\left(t\right)\dot{\overset{-}{\Theta }}+K\left(t\right)\overset{-}{\Theta }=\tau_{e} \end{array}$$

where $\overset{-}{\Theta }=\Theta -\Theta^{d}$ is defined to be an error vector of the generalized coordinates and Θ^*d*^ denotes the desired position of the generalized coordinates, *H* denotes a positive definite and symmetric constant matrix, *D*(*t*) denotes a time-varying damping matrix, and *K*(*t*) denotes a time-varying stiffness matrix. Both of *D*(*t*) and *K*(*t*) are also positive definite and symmetric. Usually, *K*(*t*) should be determined by the designated operation tasks, and *D*(*t*) should be selected to ensure the global asymptotic stability at the origin $\left(\overset{-}{\Theta },\dot{\overset{-}{\Theta }}\right)=\left(0,0\right)$ of the closed-loop system (2) when the equivalent external torque satisfies τ_*e*_ = 0. If the equivalent external torque τ_*e*_ does not equal to zero, then the origin $\left(\overset{-}{\Theta },\dot{\overset{-}{\Theta }}\right)=\left(0,0\right)$ of the closed-loop system (2) should be globally stable in Lyapunov's sense. An elegant result of designing a time-varying impedance controller of the manipulator can be stated as Lemma 1, which is an adapted result that was first presented in the literature (Kronander and Billard, 2016).

**Lemma 1**. *For the dynamic systems (1) and the target system (2), suppose the stiffness matrix K*(*t*) *is continuous, then* $\dot{K}\left(t\right)$ *is bounded, which means* $\|\dot{K}\left(t\right)\|\leq \Omega$, *where* Ω *is a positive constant. Then there exists a positive constant α and a matrix D*(*t*) *satisfying the following set of inequalities*


(3) $$\begin{array}{l} \{\begin{array}{l} \alpha >0\\ K\left(t\right)+\alpha D\left(t\right)-\alpha^{2}H>0\\ -D\left(t\right)+\alpha H<0\\ \dot{K}\left(t\right)+\alpha Ḋ\left(t\right)-2\alpha K\left(t\right)<0 \end{array} \end{array}$$

which makes the following closed-loop system


(4) $$\begin{array}{l} \{\begin{array}{l} M\left(\Theta \right)\ddot{\Theta }+C\left(\Theta ,\dot{\Theta }\right)\dot{\Theta }+N\left(\Theta \right)-\tau_{e}=\tau_{a}\\ \tau_{a}=M{\ddot{\Theta }}^{d}+C{\dot{\Theta }}^{d}+N+\left(M-H\right)\ddot{\overset{-}{\Theta }}+\left[C-D\left(t\right)\right]\dot{\overset{-}{\Theta }}\\ -K\left(t\right)\overset{-}{\Theta } \end{array} \end{array}$$

globally uniformly asymptotically stable at the origin $\left(\overset{-}{\Theta },\dot{\overset{-}{\Theta }}\right)=\left(0,0\right)$ when τ_*e*_ = 0. When τ_*e*_ ≠ 0, then the origin $\left(\overset{-}{\Theta },\dot{\overset{-}{\Theta }}\right)=\left(0,0\right)$ is globally uniformly stable.

**R****emark** **1**. By applying a Lyapunov candidate function $V\left(\dot{\overset{-}{\Theta }},\overset{-}{\Theta },t\right)=\frac{1}{2}{\left(\dot{\overset{-}{\Theta }}+\alpha \overset{-}{\Theta }\right)}^{\text{T}}H\left(\dot{\overset{-}{\Theta }}+\alpha \overset{-}{\Theta }\right)+\frac{1}{2}{\overset{-}{\Theta }}^{\text{T}}\beta \left(t\right)\overset{-}{\Theta }$ with the time-varying function definition β(*t*) = *K*(*t*) + α*D*(*t*) − α^2^*H*, it is not hard to show that the first two inequalities in (3) are used to ensure the positive definiteness of Lyapunov function $V\left(\dot{\overset{-}{\Theta }},\overset{-}{\Theta },t\right)$, and the last two inequalities in (3) can ensure the negative definiteness of $\dot{V}\left(\dot{\overset{-}{\Theta }},\overset{-}{\Theta },t\right)$. Furthermore, by proving the function $V\left(\dot{\overset{-}{\Theta }},\overset{-}{\Theta },t\right)$ is also a decrescent function, then the global uniform asymptotic stability of the closed-loop system (2) can be concluded. For the purpose of simplifying control parameters selection, in He et al. (2020) the authors presented a simple stability condition


(5) $$\begin{array}{l} D\left(t\right)=\alpha H+\varepsilon I \end{array}$$

where ε > 0 is a small constant and *I* denotes an identity matrix. Even though the damping matrix given in (5) shows certain conservatism for some applications, it is sufficient to show that the solution of the inequality group (3) exists.

**R****emark** **2**. Note that the torque controller τ_*a*_(*t*) in (4) uses acceleration feedbacks, and the dynamics model (1) is supposed to be accurate. In real world applications, these two points may not be easily achieved, since the acceleration sensors are not standard accessories for many manipulators and it is also rather difficult to accurately determine the dynamics parameters of a multi-DOF mechanical system. In the next section, it will be shown that these problems can be resolved by developing an MRAC based time-varying impedance controller.

## 3. A MRAC Based Time-varying Impedance Controller for Manipulators

For a controlled system with an adaptive controller, in general, the uniformly asymptotical stability of the closed-loop system cannot be concluded by following the same method as that provided in Remark 1. The main reason is that a parameter estimation law is also included in the closed-loop system besides a control law, such that the Lyapunov candidate function cannot be constructed as that presented in Remark 1. On the contrary, the following lemma (Slotine and Li, 1991) can be utilized to analyze the uniformly asymptotical stability of a closed-loop system with an adaptive controller.

**L****emma** **2**. *If a scalar function V*(*t*) *has the following properties, then* $\underset{\text{t}\to \infty }{\text{lim}}\dot{V}\left(t\right)\to 0$.

1. *V*(*t*) is lower bounded;
2. $\dot{V}\left(t\right)$ is negative semi-definite;
3. $\dot{V}\left(t\right)$ is uniformly continuous in time.

Now, we derive the adaptive time-varying impedance controller. First, we define a virtual velocity error vector


(6) $$\begin{array}{l} s=\dot{\overset{-}{\Theta }}+\Lambda \overset{-}{\Theta }=\dot{\Theta }-{\dot{\Theta }}^{d}+\Lambda \overset{-}{\Theta }=\dot{\Theta }-{\dot{\Theta }}_{r} \end{array}$$

where Λ ∈ ℜ^*n*×*n*^ is a symmetric and positive definite matrix, or more generally a matrix so that −Λ is Hurwitz, $\overset{-}{\Theta }\in {ℜ}^{n}$, and the virtual reference velocity ${\dot{\Theta }}_{r}\in {ℜ}^{n}$ in Equation (6) is defined as


(7) $$\begin{array}{l} {\dot{\Theta }}_{r}={\dot{\Theta }}^{d}-\Lambda \overset{-}{\Theta }. \end{array}$$

It is well known that the dynamics of a mechanical system commonly satisfies the linearly parameterized property, that is, the left-hand side of the dynamic system (1) can be expressed as the following form


(8) $$\begin{array}{l} M\left(\Theta \right)\ddot{\Theta }+C\left(\Theta ,\dot{\Theta }\right)\dot{\Theta }+N\left(\Theta \right)-\tau_{e}=\chi \left(\Theta ,\dot{\Theta },\ddot{\Theta }\right)\rho \end{array}$$

where $\chi \left(\Theta ,\dot{\Theta },\ddot{\Theta }\right)$ denotes a matrix, ρ denotes an unknown parameter vector that describes the mass properties of a mechanical system. If we replace the differential variables $\dot{\Theta }$ and $\ddot{\Theta }$ of the system (1) with the virtual reference velocity ${\dot{\Theta }}_{r}$ and its differential variable ${\ddot{\Theta }}_{r}$, then the linearly parameterized property does not change, and the resulted virtual dynamic system can also be expressed as a similar form


(9) $$\begin{array}{l} M\left(\Theta \right){\ddot{\Theta }}_{r}+C\left(\Theta ,\dot{\Theta }\right){\dot{\Theta }}_{r}+N\left(\Theta \right)-\tau_{e}=\chi \left(\Theta ,\dot{\Theta },{\dot{\Theta }}_{r},{\ddot{\Theta }}_{r}\right)\rho . \end{array}$$

By applying the linearly parameterized form Equation (9), we can obtain the following result.

**T****heorem** **1**. For the dynamic systems (1), by applying the following controller


(10) $$\begin{array}{l} \tau_{a}=\chi \left(\Theta ,\dot{\Theta },{\dot{\Theta }}_{r},{\ddot{\Theta }}_{r}\right)\hat{\rho }-K_{D}s \end{array}$$

and the following parameter estimator


(11) $$\begin{array}{l} \dot{\hat{\rho }}=-\Gamma^{-1}\chi^{\text{T}}s \end{array}$$

where *K*_*D*_ in Equation (10) is a continuous positive definite matrix, i.e., ${\dot{K}}_{D}$ is bounded, $\hat{\rho }$ denotes the estimation of ρ, and the matrix Γ in Equation (11) is also positive definite, then the origin $\left(\overset{-}{\Theta },\dot{\overset{-}{\Theta }}\right)=\left(0,0\right)$ of the closed-loop system


(12) $$\begin{array}{l} \{\begin{array}{l} M\left(\Theta \right)\ddot{\Theta }+C\left(\Theta ,\dot{\Theta }\right)\dot{\Theta }+N\left(\Theta \right)-\tau_{e}=\tau_{a}\\ \tau_{a}=\chi \left(\Theta ,\dot{\Theta },{\dot{\Theta }}_{r},{\ddot{\Theta }}_{r}\right)\hat{\rho }-K_{D}s\\ \dot{\hat{\rho }}=-\Gamma^{-1}\chi^{\text{T}}s \end{array} \end{array}$$

is globally uniformly asymptotically stable when the external loads τ_*e*_ = 0. If the external loads τ_*e*_ ≠ 0, the origin $\left(\overset{-}{\Theta },\dot{\overset{-}{\Theta }}\right)=\left(0,0\right)$ of the system Equation (12) is globally uniformly stable in the Lyapunov's sense.

PROOF. Let us define $\overset{-}{\rho }=\hat{\rho }-\rho$ to be an error vector of the parameter estimates $\hat{\rho }$ and select a Lyapunov candidate function


(13) $$\begin{array}{l} V\left(t\right)=\frac{1}{2}\left(s^{\text{T}}Ms+{\overset{-}{\rho }}^{\text{T}}\Gamma \overset{-}{\rho }\right). \end{array}$$

By using the definition of the virtual velocity error vector given by Equation (6), the time derivative of Equation (13) can be given as


(14) $$\begin{array}{l} \begin{array}{l} \dot{V}\left(t\right)=s^{\text{T}}Mṡ+\frac{1}{2}s^{\text{T}}Ṁs+{\overset{-}{\rho }}^{\text{T}}\Gamma \dot{\overset{-}{\rho }}=s^{\text{T}}\left(M\ddot{\Theta }-M{\ddot{\Theta }}_{r}\right)+\frac{1}{2}s^{\text{T}}Ṁs\\ +{\overset{-}{\rho }}^{\text{T}}\Gamma \dot{\overset{-}{\rho }}. \end{array} \end{array}$$

Since Ṁ − 2*C* is a skew-symmetric matrix (Murray et al., 1994), which means that (Ṁ − 2*C*)^T^ = −(Ṁ − 2*C*), we have


(15) $$\begin{array}{l} {Ṁ}^{\text{T}}+Ṁ=2C^{\text{T}}+2C \end{array}$$

and since *M* is a symmetric and positive definite (Murray et al., 1994), which means that *M* = *M*^T^, then from Equation (15) we can get


(16) $$\begin{array}{l} {Ṁ}^{\text{T}}+Ṁ=2Ṁ=2C^{\text{T}}+2C \end{array}$$

So


(17) $$\begin{array}{l} Ṁ=C+C^{\text{T}} \end{array}$$

By using the equation above, Equation (14) can be written as


(18) $$\begin{array}{l} \dot{V}\left(t\right)=s^{\text{T}}\left(M\ddot{\Theta }-M{\ddot{\Theta }}_{r}\right)+\frac{1}{2}s^{\text{T}}\left(C+C^{\text{T}}\right)s+{\overset{-}{\rho }}^{\text{T}}\Gamma \dot{\overset{-}{\rho }}. \end{array}$$

Referring to the dynamics Equation (1), it is easy to obtain


(19) $$\begin{array}{l} M\ddot{\Theta }=\tau_{a}-C\dot{\Theta }-N+\tau_{e} \end{array}$$

and from (6) we can obtain


(20) $$\begin{array}{l} \dot{\Theta }=s+{\dot{\Theta }}_{r}. \end{array}$$

Substituting Equations (20) into (19) and then bringing Equations (19) into (18), it can be shown that


(21) $$\begin{array}{l} \begin{array}{l} \dot{V}\left(t\right)=s^{\text{T}}\left[\tau_{a}-M{\ddot{\Theta }}_{r}-C\left(s+{\dot{\Theta }}_{r}\right)-N+\tau_{e}\right]+\frac{1}{2}s^{\text{T}}\left(C+C^{\text{T}}\right)\\ s+{\overset{-}{\rho }}^{\text{T}}\Gamma \dot{\overset{-}{\rho }}\\ =s^{\text{T}}\left[\tau_{a}-M{\ddot{\Theta }}_{r}-C{\dot{\Theta }}_{r}-N+\tau_{e}\right]+{\overset{-}{\rho }}^{\text{T}}\Gamma \dot{\overset{-}{\rho }}. \end{array} \end{array}$$

Due to $\overset{-}{\rho }=\hat{\rho }-\rho$ and ρ is a constant for any manipulator system, we have $\dot{\overset{-}{\rho }}=\dot{\hat{\rho }}$. Therefore, Equation (21) follows that


(22) $$\begin{array}{l} \dot{V}\left(t\right)=s^{\text{T}}\left[\tau_{a}-M{\ddot{\Theta }}_{r}-C{\dot{\Theta }}_{r}-N+\tau_{e}\right]+{\overset{-}{\rho }}^{\text{T}}\Gamma \dot{\hat{\rho }}. \end{array}$$

By applying the linearly parameterized form Equation (9), Equation (22) can be expressed as


(23) $$\begin{array}{l} \dot{V}\left(t\right)=s^{\text{T}}\left[\tau_{a}-\chi \left(\Theta ,\dot{\Theta },{\dot{\Theta }}_{r},{\ddot{\Theta }}_{r}\right)\rho \right]+{\overset{-}{\rho }}^{\text{T}}\Gamma \dot{\hat{\rho }}. \end{array}$$

If we adopt the controller Equation (10), it is straightforward that the Equation (23) can be rewritten as


(24) $$\begin{array}{l} \begin{array}{l} \dot{V}\left(t\right)=s^{\text{T}}\left[\chi \hat{\rho }-K_{D}s-\chi \rho \right]+{\overset{-}{\rho }}^{\text{T}}\Gamma \dot{\hat{\rho }}=s^{\text{T}}\chi \overset{-}{\rho }-s^{\text{T}}K_{D}s+{\overset{-}{\rho }}^{\text{T}}\Gamma \dot{\hat{\rho }}. \end{array} \end{array}$$

By using the parameter estimator Equation (11), which is given as $\dot{\hat{\rho }}=-\Gamma^{-1}\chi^{\text{T}}s$, then we can obtain


(25) $$\begin{array}{l} \dot{V}\left(t\right)=-s^{\text{T}}K_{D}s\leq 0 \end{array}$$

since *K*_*D*_ is positive definite. This implies *V*(*t*) ≤ *V*(0), and therefore, both of the vectors *s* and $\overset{-}{\rho }$ are bounded [see Equation (13)]. To observe the uniform continuity of the function $\dot{V}\left(t\right)$, we calculate the second order differential function of *V*(*t*), and it can be written as


(26) $$\begin{array}{l} \ddot{V}\left(t\right)=-2s^{\text{T}}K_{D}ṡ-s^{\text{T}}{\dot{K}}_{D}s. \end{array}$$

See definition Equation (6), it shows the vector *s* is smooth. On the other hand, the differential matrix ${\dot{K}}_{D}$ is supposed to be bounded. Then we can conclude that $\ddot{V}\left(t\right)$ is bounded. According to Lemma 2, we can get $\underset{\text{t}\to \infty }{\text{lim}}\dot{V}\left(t\right)\to 0$, which means *s* → 0 as *t* → ∞. It is obvious that ṡ is bounded.

On the surface *s* = 0, referring to the definition $s=\dot{\overset{-}{\Theta }}+\Lambda \overset{-}{\Theta }$, we can conclude the origin $\left(\overset{-}{\Theta },\dot{\overset{-}{\Theta }}\right)=\left(0,0\right)$ of the closed-loop system Equation (12) is uniformly asymptotically stable since −Λ is Hurwitz. In addition, the function *V*(*t*) is unbounded, thus the stability of the closed-loop system is globally effective.

**R****emark** **3**. Theorem 1 shows that both the control law Equation (10) and the parameter estimator (11) only use state feedback $s=\dot{\overset{-}{\Theta }}+\Lambda \overset{-}{\Theta }$. This is helpful for improving the feasibility of the controller in real world applications. In particular, the adaptive controller does not need an accurate dynamic model, thus better robust stability of the closed-loop system Equation (12) could be expected.

**R****emark** **4**. Even though Theorem 1 gives an adaptive controller for the dynamic system Equation (1), so far the adaptive controller is not related to the time-varying impedance control issues of the manipulators. By using the following result, we can get that the time-varying impedance control problems can be resolved under the adaptive control strategy.

**T****heorem** **2**. If the control parameters Λ and *K*_*D*_ of the adaptive controller Equation (10) are chosen as


(27) $$\begin{array}{l} \Lambda =\gamma M^{-1},K_{D}=\frac{1}{\gamma }K\left(t\right)M-C \end{array}$$

where γ > 0 is a constant, then the origin $\left(\overset{-}{\Theta },\dot{\overset{-}{\Theta }}\right)=\left(0,0\right)$ of the closed-loop system Equation (12) is globally uniformly stable in Lyapunov's sense.

PROOF. By subtracting Equation (9) from Equation (1), we have


(28) $$\begin{array}{l} Mṡ+Cs=\tau_{a}-\chi \rho \end{array}$$

where $s=\dot{\Theta }-{\dot{\Theta }}_{r}$ is considered. Then, substituting the adaptive control law Equation (10) into Equation (28), we can obtain that


(29) $$\begin{array}{l} Mṡ+\left(C+K_{D}\right)s=\chi \overset{-}{\rho } \end{array}$$

where $\overset{-}{\rho }=\hat{\rho }-\rho$ is considered. Since the vector also satisfies the relationship $s=\dot{\overset{-}{\Theta }}+\Lambda \overset{-}{\Theta }$, we can obtain


(30) $$\begin{array}{l} M\ddot{\overset{-}{\Theta }}+\left(M\Lambda +C+K_{D}\right)\dot{\overset{-}{\Theta }}+\left(C+K_{D}\right)\Lambda \overset{-}{\Theta }=\chi \overset{-}{\rho }. \end{array}$$

According to Equation (27), if we select Λ = γ*M*^−1^ and $K_{D}=\frac{1}{\gamma }K\left(t\right)M-C$, then Equation (30) can be written as


(31) $$\begin{array}{l} M\ddot{\overset{-}{\Theta }}+D\left(t\right)\dot{\overset{-}{\Theta }}+K\left(t\right)\overset{-}{\Theta }=\chi \overset{-}{\rho } \end{array}$$

where


(32) $$\begin{array}{l} \begin{array}{l} D\left(t\right)=M\Lambda +C+K_{D}=\gamma I+\frac{1}{\gamma }K\left(t\right)M. \end{array} \end{array}$$

Comparing Equation (32) with Equation (5), it shows the damping matrix given by (32) satisfies the stability condition Equation (5) if we select $\alpha =\frac{1}{\gamma }K\left(t\right)$ and ε = γ. In addition, on the basis of Theorem 1, under the control law (10) and the parameter estimator Equation (11), the error vector $\overset{-}{\rho }$ is bounded. According to Lemma 1, the origin $\left(\overset{-}{\Theta },\dot{\overset{-}{\Theta }}\right)=\left(0,0\right)$ of the closed-loop system Equation (31) is globally uniformly asymptotically stable when $\overset{-}{\rho }=0$. If the error vector $\overset{-}{\rho }\neq 0$, the origin $\left(\overset{-}{\Theta },\dot{\overset{-}{\Theta }}\right)=\left(0,0\right)$ of the system Equation (31) is globally uniformly stable in Lyapunov's sense.

**R****emark** **5**. It is worth noting that, in Equation (2), the inertial matrix *H* is generally different from the inertial matrix *M*, so that the term $\left(M-H\right)\ddot{\overset{-}{\Theta }}$ is appeared in the controller Equation (4), and then an accelerated feedback or sensing the external loads τ_*e*_ is necessary. If we select *H* = *M*, the closed-loop system Equation (4) is given by


(33) $$\begin{array}{l} M\ddot{\overset{-}{\Theta }}+D\left(t\right)\dot{\overset{-}{\Theta }}+K\left(t\right)\overset{-}{\Theta }=\tau_{e} \end{array}$$

which is very similar to the adaptive control law based closed-loop system Equation (31). However, if the dynamics model Equation (1) is not accurate, then the error terms $\Delta M\left(\Theta \right)\ddot{\Theta }$, $\Delta C\left(\Theta ,\dot{\Theta }\right)\dot{\Theta }$, and Δ*N*(Θ) will appear in the closed-loop system Equation (4), as well as in the system Equation (33), so that some more complex robust controllers have to be used to overcome the effects caused by the un-modeled errors for guaranteeing the stability of the closed-loop system Equation (4). On the contrary, the adaptive control law Equation (10) has considered the un-modeled error and updated the virtual reference model $\chi \left(\Theta ,\dot{\Theta },{\dot{\Theta }}_{r},{\ddot{\Theta }}_{r}\right)\hat{\rho }$ in the controller Equation (10) online by using the parameter estimator Equation (11). This makes the virtual velocity vectors *s* and the parameters errors $\overset{-}{\rho }$ be bounded, and finally, the virtual velocity vectors *s* → 0 as *t* → ∞. On the surface $s=\dot{\overset{-}{\Theta }}+\Lambda \overset{-}{\Theta }=0$, the stability of the state $\left(\overset{-}{\Theta },\dot{\overset{-}{\Theta }}\right)$ of the closed-loop system is ensured by the Hurwitz matrix −Λ. Thus, the two problems mentioned in Remark 2 can be resolved or relaxed by using the MRAC based time-varying impedance controller.

**R****emark** **6**. It is also worth noting that, in general, the external loads τ_*e*_ cannot be estimated by using the linearly parameterized form Equation (9). Thus, for some accurate force tracking control tasks, the linearly parameterized form Equation (9) should be changed as


(34) $$\begin{array}{l} M\left(\Theta \right){\ddot{\Theta }}_{r}+C\left(\Theta ,\dot{\Theta }\right){\dot{\Theta }}_{r}+N\left(\Theta \right)=\chi \left(\Theta ,\dot{\Theta },{\dot{\Theta }}_{r},{\ddot{\Theta }}_{r}\right)\rho \end{array}$$

then under the control law Equation (10) and the parameter estimator Equation (11), the closed-loop system can be given as


(35) $$\begin{array}{l} M\ddot{\overset{-}{\Theta }}+D\left(t\right)\dot{\overset{-}{\Theta }}+K\left(t\right)\overset{-}{\Theta }=\chi \overset{-}{\rho }+\tau_{e} \end{array}$$

where the control parameter selection Equation (27) is considered. Since the right side of Equation (35) is bounded under control law Equation (10) with the parameter estimator Equation (11), the origin $\left(\overset{-}{\Theta },\dot{\overset{-}{\Theta }}\right)=\left(0,0\right)$ of the system Equation (35) is still globally uniformly stable in the Lyapunov's sense. However, the error term $\chi \overset{-}{\rho }$ on the right side of Equation (35) will cause certain force tracking errors. Thus, for accurate force tracking control tasks, measurement of external loads is required, and the MRAC based control law should be changed as


(36) $$\begin{array}{l} \{\begin{array}{l} \tau_{a}=\chi \left(\Theta ,\dot{\Theta },{\dot{\Theta }}_{r},{\ddot{\Theta }}_{r}\right)\hat{\rho }-K_{D}s-\tau_{e}\\ \dot{\hat{\rho }}=-\Gamma^{-1}\chi^{\text{T}}s \end{array} \end{array}$$

then the closed-loop system Equation (35) will changed to that same as Equation (31).

## 4. Numerical Simulations

To test the feasibility of the proposed adaptive time-varying impedance controller, a model-uncertain planar 2R manipulator is adopted as the plant. Suppose the mass of two links are *m*_1_ and *m*_2_, respectively, the inertia of two links is *I*_1_ and *I*_2_, respectively, the length of two links is *L*_1_ and *L*_2_, respectively, the distance between the mass center of links and joint axes are *L*_*c*1_ and *L*_*c*2_, respectively, the dynamic equation of planar 2R manipulator can be given as


(37) $$\begin{array}{l} \left[\begin{array}{cc} m_{11} & m_{12}\\ m_{21} & m_{22} \end{array}\right]\left[\begin{array}{c} {\ddot{\theta }}_{1}\\ {\ddot{\theta }}_{2} \end{array}\right]+\left[\begin{array}{cc} c_{11} & c_{12}\\ c_{21} & c_{22} \end{array}\right]\left[\begin{array}{c} {\dot{\theta }}_{1}\\ {\dot{\theta }}_{2} \end{array}\right]=\left[\begin{array}{c} \tau_{1}\\ \tau_{2} \end{array}\right] \end{array}$$

where θ_1_ and θ_2_ are the joint angles of the two links, *m*_11_ = ρ_1_ + 2ρ_3_ cos θ_2_, *m*_12_ = ρ_2_ + ρ_3_ cos θ_2_, *m*_21_ = *m*_12_, *m*_22_ = ρ_2_, $c_{11}=-\rho_{3}\sin\theta_{2}{\dot{\theta }}_{2}$, $c_{12}=-\rho_{3}\sin\theta_{2}\left({\dot{\theta }}_{1}+{\dot{\theta }}_{2}\right)$, $c_{21}=\rho_{3}\sin\theta_{2}{\dot{\theta }}_{1}$, and *c*_22_ = 0 with $\rho_{1}=I_{1}+m_{1}L_{c1}^{2}+I_{2}+m_{2}\left(L_{1}^{2}+L_{c2}^{2}\right)$, $\rho_{2}=I_{2}+m_{2}L_{c2}^{2}$, and ρ_3_ = *m*_2_*L*_1_*L*_*c*2_. For the planar 2R manipulator, the linearly parameterized form Equation (34) can be expressed as


(38) $$\begin{array}{l} \chi \rho =\left[\begin{array}{ccc} \chi_{11} & \chi_{12} & \chi_{13}\\ \chi_{21} & \chi_{22} & \chi_{23} \end{array}\right]\left[\begin{array}{c} \rho_{1}\\ \rho_{2}\\ \rho_{3} \end{array}\right] \end{array}$$

where $\chi_{11}={\ddot{\theta }}_{1r}$, $\chi_{12}={\ddot{\theta }}_{2r}$, $\chi_{13}=\left(2{\ddot{\theta }}_{1r}+{\ddot{\theta }}_{2r}\right)\cos\theta_{2}-\left({\dot{\theta }}_{2}{\dot{\theta }}_{1r}+{\dot{\theta }}_{1}{\dot{\theta }}_{2r}+{\dot{\theta }}_{2}{\dot{\theta }}_{2r}\right)\sin\theta_{2}$, χ_21_ = 0, $\chi_{22}={\ddot{\theta }}_{1r}+{\ddot{\theta }}_{2r}$, $\chi_{23}={\ddot{\theta }}_{1r}\cos\theta_{2}+{\dot{\theta }}_{1}{\dot{\theta }}_{1r}\sin\theta_{2}$. In the simulation, the control task is described as


(39) $$\begin{array}{l} \{\begin{array}{cc} \theta_{1}^{d}\left(t\right)=\frac{\pi }{4}+\frac{\pi }{6}\sin\left(2\pi t\right) & t\leq 6s\\ \theta_{2}^{d}\left(t\right)=-\frac{\pi }{4}+\frac{\pi }{5}\sin\left(2\pi t\right) & t\leq 6s\\ \theta_{1}^{d}\left(t\right)=\frac{\pi }{4},\theta_{2}^{d}\left(t\right)=-\frac{\pi }{4} & t>6s \end{array} \end{array}$$

and


(40) $$\begin{array}{l} \{\begin{array}{cc} \tau_{e}={\left[\begin{array}{cc} 0 & 0 \end{array}\right]}^{\text{T}} & t\leq 10s\\ \tau_{e}={\left[\begin{array}{cc} 5 & -5 \end{array}\right]}^{\text{T}} & 10s<t\leq 14s\\ \tau_{e}={\left[\begin{array}{cc} 0 & 0 \end{array}\right]}^{\text{T}} & t>14s \end{array}. \end{array}$$

**Figure 1:**
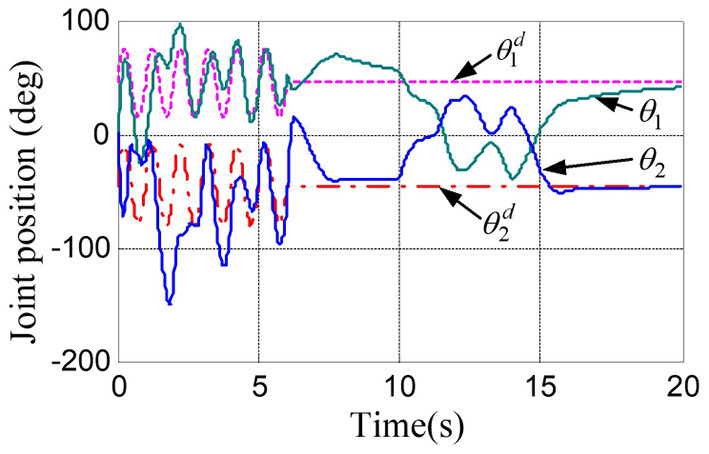
Responses of the joint position and their desired trajectories.

**Figure 2:**
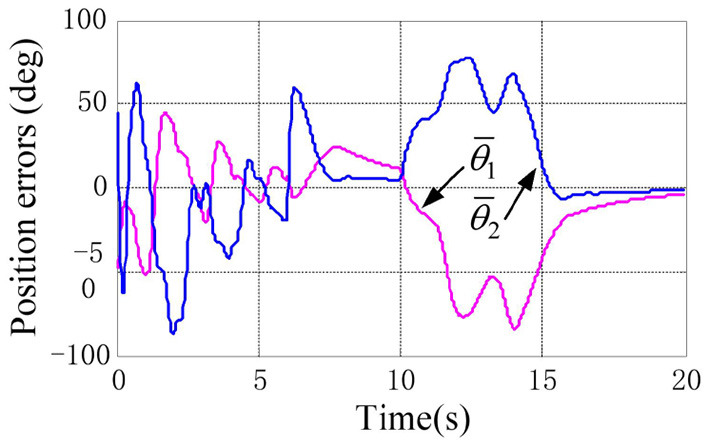
Responses of the joint position errors.

**Figure 3:**
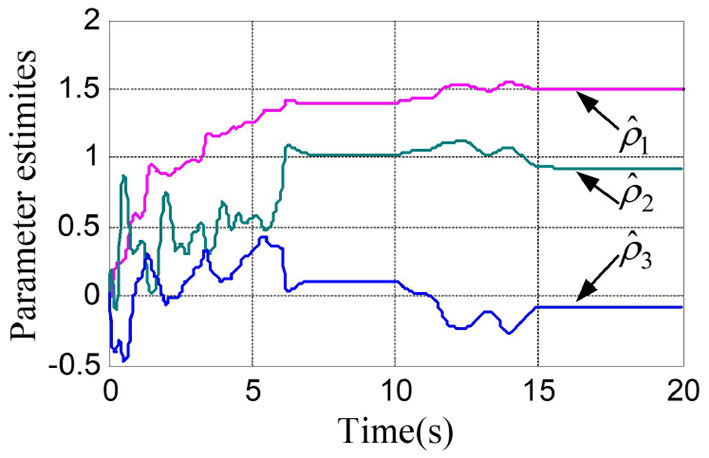
Trajectories of the parameter estimates.

**Figure 4:**
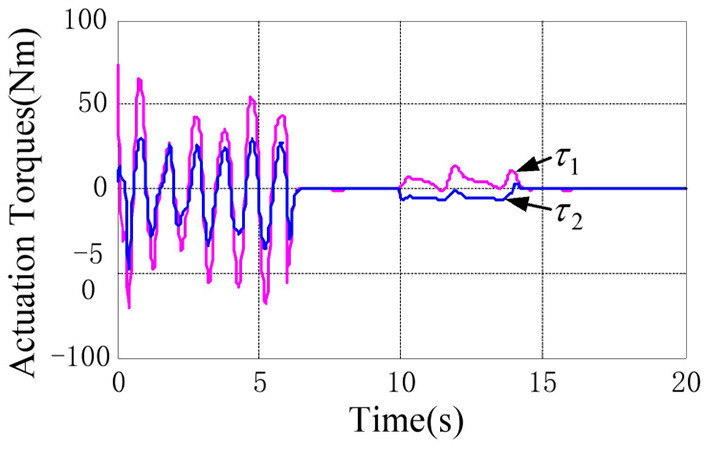
Actuation torques of the 2R manipulator during the control task.

**Figure 5:**
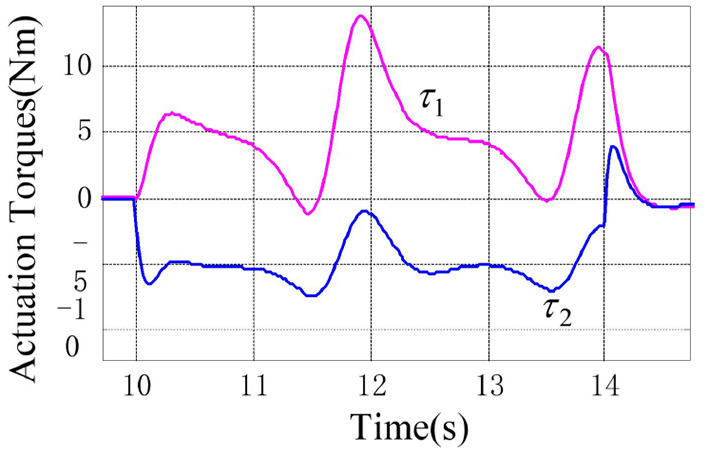
A local enlarged drawing of the actuation torques when the external loads are acting.

The physical parameters of the manipulator are shown in Table 1, and the control parameters are shown in Table 2, then the response results of the closed-loop system Equation (35) are plotted in the Figures 1–**5**.

**Table 1:** Physical parameters of the planar 2R manipulator.

**Parameter** **Symbols**	**Initial value** **used in $\hat{\rho }$**	**Actual value** **of the plant**	**Physical** **Units**
*m*_1_	0	2.0	kg
*m*_2_	0	2.0	kg
*L*_1_	0	0.5	m
*L*_2_	0	0.6	m
*L*_*c*1_	0	0.3	m
*L*_*c*2_	0	0.4	m
$I_{1}=m_{1}L_{c1}^{2}$	0	0.18	Kg · m^2^
$I_{2}=m_{2}L_{c2}^{2}$	0	0.32	Kg · m^2^

**Table 2:** Control parameters of the adaptive controller.

**Parameters**	**Symbols**	**Values**	**Physical** **Units**
Coefficient	γ	0.04	/
Inertial matrix	*M*	Given by (37)	Kg · m^2^
Coefficient matrix	Λ	γ*M*^−1^	(Kg · m^2^)^−1^
Coefficient matrix	Γ	80*I*	/
Desired stiffness matrix	*K*(*t*)	$\left[\begin{array}{cc} 5+4\sin\left(\pi t\right) & 0\\ 0 & 5-4\cos\left(\pi t\right) \end{array}\right]$	Nm/rad
Coefficient matrix	*K*_*D*_	$\frac{1}{\gamma }K\left(t\right)M-C$	/
Desired damping matrix	*D*(*t*)	$\gamma I+\frac{1}{\gamma }K\left(t\right)M$	Nm/rad/s

According to the numerical simulation results, even though the physical parameters of the plant are supposed to be zero at the initial moment (see Figure 3 and Table 1), it shows that system Equation (35) is uniformly stable for the controlled planar 2R manipulator. From Figures 1, 2, it can be seen that the joint trajectory tracking errors are bounded and converge to zero when the trajectory tracking task is switched to a stabilization task after the time is larger than 6s. Meanwhile, from Figure 3, it is observed that the parameter estimates $\hat{\rho }$ are changed to constant values, and from Figure 4, one sees that the actuation torques converge to zero after the desired joint trajectories $\theta_{i}^{d}\left(t\right)$ are constants. When the time is falling in the interval *t* ∈ (10, 14](s), there are non-zero external loads $\tau_{e}={\left[5-5\right]}^{\text{T}}$ acting on the joints, and then the joint angles demonstrate large deviations (as shown in Figure 2) due to the small given closed-loop stiffness *K*(*t*) (as shown in Table 2). Since the desired joint stiffness *K*(*t*) is time-varying, the joint position deviations are varying even though the external loads τ_*e*_ are constant. Figure 5 shows a local enlarged drawing of the actuation torques during τ_*e*_ ≠ 0. It is observed that the average values of the actuation torques happen to be $\tau_{a}\approx {\left[5-5\right]}^{\text{T}}$, since the planar 2R manipulator moves in the horizontal plane [see Equation (37) where the gravity of the manipulator is not considered here], then the actuation torques τ_*a*_ should balance the external loads τ_*e*_. However, due to the desired time-varying stiffness *K*(*t*), the parameter estimates $\hat{\rho }$ show certain fluctuations (such that $\overset{-}{\rho }\neq 0$), then the error term $\chi \overset{-}{\rho }$ shown in Equation (35) causes the actuation torques τ_*a*_ to show certain fluctuations. The selection of controller parameters γ and Γ affect the performance of the system. We make a performance analysis of the closed-loop control system with different parameters γ and Γ. We found that with other conditions unchanged, when Γ increases within a certain range, the root-mean-square error (RMSE) of the joint position will increase, and the peak value of the error $\overset{-}{\Theta }$ will also increase, while the RMSE of control torque will decrease. When γ is too large or too small, the performance of the control system will deteriorate. Therefore, the state-independent property allows us to tune the controller parameters offline in advance through simulation, which lays a good foundation for ensuring the performance of the robot.

**Figure 6:**
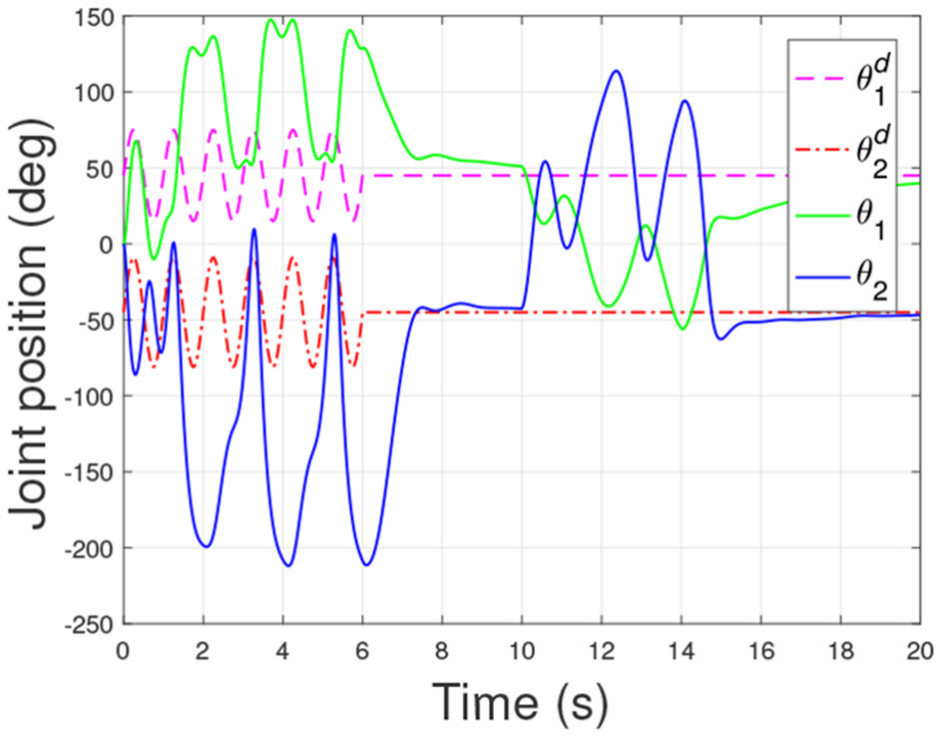
Responses of the joint position and their desired trajectories of the comparison method.

**Figure 7:**
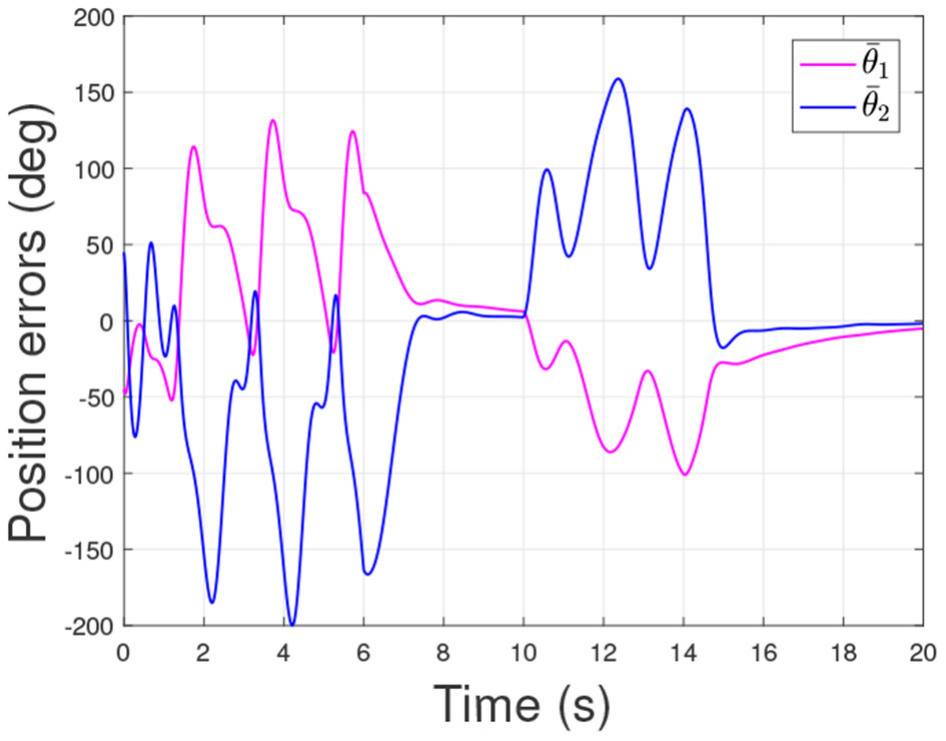
Responses of the joint position errors of the comparison method.

**Figure 8:**
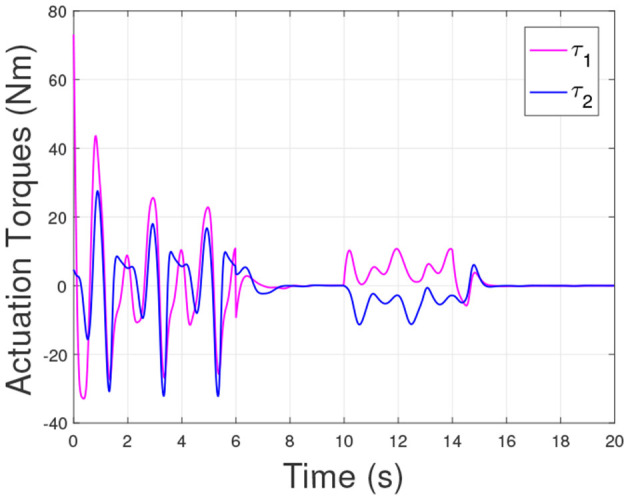
Actuation torques of the 2R manipulator during the control task of the comparison method.

In order to verify the effectiveness of the controller proposed in this article, we also compared it with the controller in He et al. (2020). Under the same initial conditions and parameters as the proposed controller, the simulation of the comparison controller is carried out, and the response results of the closed-loop system under the comparison controller are shown in Figures 6–8. Comparing Figures 2, 7, we can get that the RMSE of the joint position under the proposed controller in Figure 2 is 0.416 and 0.494, while the RMSE of the joint position under the comparison controller in Figure 7 is 0.865 and 1.337. Then, it can be concluded that the controller proposed in this article can better realize the trajectory tracking control with higher accuracy. From the simulation results, we can also get that the proposed controller has a smaller peak error. All these simulation results verify the effectiveness of the controller proposed in this article.

## 5. Conclusion

Under the design frame of an MRAC based control system, a time-varying impedance controller is proposed for manipulators with uncertain structure parameters. We show that the proposed controller does not need to use acceleration-based feedback or measurement of the external loads, and the adaptive controller can tolerate considerable structure parameter errors. By employing a Lyapunov-like stability analysis approach, the globally uniform stability of the time-varying closed-loop system is analyzed, and a simple controller parameters selection approach is presented. Through a planar 2R manipulator, the feasibility of the proposed control method is verified by some numerical simulations. Our future work will focus on the anti-interference ability of the proposed controller.

## Data Availability Statement

The original contributions presented in the study are included in the article/supplementary material, further inquiries can be directed to the corresponding author.

## Author Contributions

XL: conceptualization, visualization, and supervision. GH: methodology. TS: software. SL and QZ: validation. CH: formal analysis. LZ: investigation. JY: resources. ZZ: writing-original draft preparation. TS and GH: writing-review and editing. JZ: project administration. XL and TS: funding acquisition. All authors have read and agreed to the published version of the manuscript. All authors agree to be accountable for the content of the work.

## Funding

This work is supported by the National Key R&D Program of China under Grants 2019YFB1309603 and 2020AAA0105801, the Natural Science Foundation of Beijing under Grants L202020 and 4204097, the Natural Science Foundation of China under Grant 62003005, 62103007 and 51775002, Beijing Municipal Education Commission under Grant KM202110009009 and KZ202010009015, China Postdoctoral Science Foundation under Grant 2021M693404, Fundamental Research Funds for Beijing Municipal Universities, Yuyou Talent Support Project of North China University of Technology, and open research fund of the State Key Laboratory for Management and Control of Complex Systems under Grant 20210103.

## Conflict of Interest

ZZ was employed by Beijing Aerospace Measurement & Control Technology Co., Ltd. The remaining authors declare that the research was conducted in the absence of any commercial or financial relationships that could be construed as a potential conflict of interest.

## Publisher's Note

All claims expressed in this article are solely those of the authors and do not necessarily represent those of their affiliated organizations, or those of the publisher, the editors and the reviewers. Any product that may be evaluated in this article, or claim that may be made by its manufacturer, is not guaranteed or endorsed by the publisher.
